# Interferon-γ-Inducible Chemokines as Prognostic Markers for Lung Cancer

**DOI:** 10.3390/ijerph18179345

**Published:** 2021-09-04

**Authors:** Keu-Sung Lee, Wou-Young Chung, Ji-Eun Park, Yun-Jung Jung, Joo-Hun Park, Seung-Soo Sheen, Kwang-Joo Park

**Affiliations:** Department of Pulmonology and Critical Care Medicine, Ajou University School of Medicine, Suwon 16499, Korea; plator@aumc.ac.kr (K.-S.L.); biscut@ajou.ac.kr (W.-Y.C.); petitprince012@ajou.ac.kr (J.-E.P.); tomato81@aumc.ac.kr (Y.-J.J.); lungmd@ajou.ac.kr (J.-H.P.); ssheen@ajou.ac.kr (S.-S.S.)

**Keywords:** cell-mediated immunity, lung cancer, interferon-γ, CXCR3 ligand, biomarker, prognosis

## Abstract

Interferon (IFN)-γ-inducible chemokines in the CXCR3/ligand axis are involved in cell-mediated immunity and play a significant role in the progression of cancer. We enrolled patients with lung cancer (*n* = 144) and healthy volunteers as the controls (*n* = 140). Initial blood samples were collected and concentrations of IFN-γ and IFN-γ-inducible chemokines CXCL9, CXCL10, and CXCL11 were measured using enzyme-linked immunosorbent assay. Of patients with lung cancer, 125 had non-small cell lung cancer (NSCLC) and 19 had small cell lung cancer. The area under the curve (AUC) (95% CI) of CXCL9 was 0.83 (0.80–0.89) for differentiating lung cancer patients from controls. The levels of all the markers were significantly higher in NSCLC patients with stage IV than in those with stages I–III. A Kaplan-Meier survival analysis showed that NSCLC cancer patients with higher levels of all markers showed poorer survival than those with lower levels. In Cox multivariate analysis of patients with NSCLC, independent prognostic factors for overall survival were CXCL9 and CXCL11. CXCL9 was the only independent prognostic factor for cancer-specific survival. Serum IFN-γ-inducible chemokines may be useful as clinical markers of metastasis and prognosis in NSCLC, and CXCL9 levels showed the most significant results.

## 1. Introduction

The immune system plays a significant role in cancer development and progression by influencing the tumor microenvironment. Immune responses to malignancy can be utilized as a target for treatment as well as for diagnostic and prognostic markers [1,2].

Chemokines play a pivotal role in immunologic reactions through the recruitment of various immune cells. Chemokines influence the development and progression of malignant tumors by trafficking activated immune cells, controlling inflammation, and regulating angiogenesis [3,4].

The interferon (IFN)-γ-inducible chemokines CXCL9 (monokine induced by IFN-γ, MIG), CXCL10 (IFN-γ-inducible 10-kDa protein, IP-10), and CXCL11 (IFN-inducible T-cell α chemoattractant, I-TAC) act as the main ligands for CXCR3, which is expressed on various cell types including T lymphocytes, malignant cells, and mesenchymal cells [5,6]. IFN-γ stimulates the release of IFN-γ-inducible chemokines from endothelial cells, fibroblasts, mononuclear cells, and tumor cells. The interaction between CXCR3 and its ligands activates cell-mediated immunity through the recruitment of CD4+ T helper type 1 (Th1) lymphocytes and NK cells [6,7].

The action of the CXCR3/ligand axis can result in different consequences depending on the types of cells and receptors located in malignant tissues [7,8,9]. As a part of the normal homeostatic response, CXCR3-harboring immune cells are activated by IFN-γ-inducible chemokines to execute anti-tumor activities. However, this cytotoxic immune reaction can be suppressed in cancer tissues by several evasive action mechanisms, including the activation of regulatory T-cells, which suppress cellular immune reactions [10], tumor-associated macrophage M2 polarization [11], and activation of immune regulatory checkpoint molecules [12]. CXCR3 expressed on cancer cells has unique action mechanisms. In particular, CXCR3-A, a predominant isoform in cancer cells, may promote proliferation, chemotaxis, cell migration, and invasion of cancer cells, while simultaneously down-regulating the anti-tumor activity of isoform CXCR3-B [13,14,15]. As a result, the overexpression of CXCR3 and IFN-γ-inducible chemokines in cancer tissues has been shown to be associated with cancer progression and poor prognosis [16,17,18,19].

High levels of circulating IFN-γ-inducible chemokines have been associated with a poor prognosis in many types of cancers, including colorectal cancer [20], osteosarcoma [21], multiple myeloma [22], nasopharyngeal cancer [23], and oral cavity carcinoma [24]. This relationship may imply that IFN-γ-inducible chemokines show greater affinity for CXCR3 on cancer cells over Th1 immune cells.

Studies on the role of peripheral blood IFN-γ-inducible chemokines in lung cancer are very limited. Increased blood levels of CXCL9 have been associated with an increased risk of lung cancer [25]. A previous study found that levels of plasma IFN-γ-inducible chemokines in non-small cell lung cancer (NSCLC) patients were higher in patients with metastasis versus patients with stages I–II tumors [26]. In addition, the concentration gradients of CXCL9 and CXCL10 from peripheral blood to the intra-tumoral environment were shown to be correlated with prognosis in patients with NSCLC [27].

Here, we evaluated the clinical implications and prognostic significance of IFN-γ-inducible chemokines by measuring levels of CXCL9, CXCL10, and CXCL11 and their inducer IFN-γ in the peripheral blood of patients with lung cancer.

## 2. Materials and Methods

### 2.1. Study Participants

We enrolled patients who were treated for lung cancer at Ajou University Hospital between 2002 and 2012. For control subjects, age- and sex-matched healthy volunteers were enrolled in the study between 2010 and 2012. The diagnosis of lung cancer was confirmed by histological examinations of either biopsy or cytological specimens.

NSCLC was staged using the Eighth Edition of the TNM Classification for Lung Cancer [28], and small cell lung cancer (SCLC) was staged as either limited or extensive [29]. The staging procedure included bronchoscopy, chest radiography, computed tomography (CT) scans of the chest, ultrasonography of the abdomen, magnetic resonance imaging of the brain, and positron emission tomography (PET). To simplify the statistical analysis, the staging of NSCLC was limited to I, II, III, and IV due to the limited number of patients.

This study was approved by the Institutional Review Board of Ajou University Hospital (MED-SMP-12). All patients provided informed, signed consent prior to sample collection.

### 2.2. Sample Collection and Processing

Initial blood samples were taken at the time of diagnosis prior to any therapeutic interventions. The collection tubes were centrifuged at 25 °C for 10 min at 1300× *g*. Following centrifugation, the sera were immediately frozen at −70 °C until further analysis.

### 2.3. Measurement of Cytokines

Enzyme-linked immunosorbent assays (ELISAs) were used to measure CXCL9, CXCL10, CXCL11, and IFN-γ (Quantikine; R&D Systems, Minneapolis, MN, USA) in the sera samples.

### 2.4. Statistical Analysis

SPSS ver. 22 for Windows (IBM, Armonk, NY, USA) was used for all statistical analyses. All values are presented as medians (interquartile range), except for the survival period, for which means ± standard error was used. The values did not fit a standard distribution, so a nonparametric analysis was performed. The Mann–Whitney U test was used to compare between two groups, and the Kruskal–Wallis test was used to compare multiple groups. Receiver operating characteristic (ROC) analysis was conducted to evaluate the predictive performance of the markers. Area under the curve (AUC) was obtained, and optimal cut-off values were determined using the Youden index to maximize the sum of sensitivity and specificity. Survival curves were plotted using the Kaplan–Meier method, and the significance of differences between groups was analyzed using the log-ranks test. A univariate Cox proportional hazards model analysis was performed to identify significant prognostic variables, which were further assessed by constructing a multivariate Cox model to determine independent prognostic factors. A *p*-value < 0.05 was considered statistically significant.

## 3. Results

### 3.1. Demographic Data

Our study enrolled 144 patients diagnosed with lung cancer, consisting of NSCLC (*n* = 125) and SCLC (*n* = 19). As control subjects, 140 healthy volunteers were enrolled. Baseline demographic data are presented in Table 1. 

### 3.2. Serum IFN-γ-Inducible Chemokines and IFN-γ Levels in Lung Cancer Patients Compared to Controls

**Figure 1:**
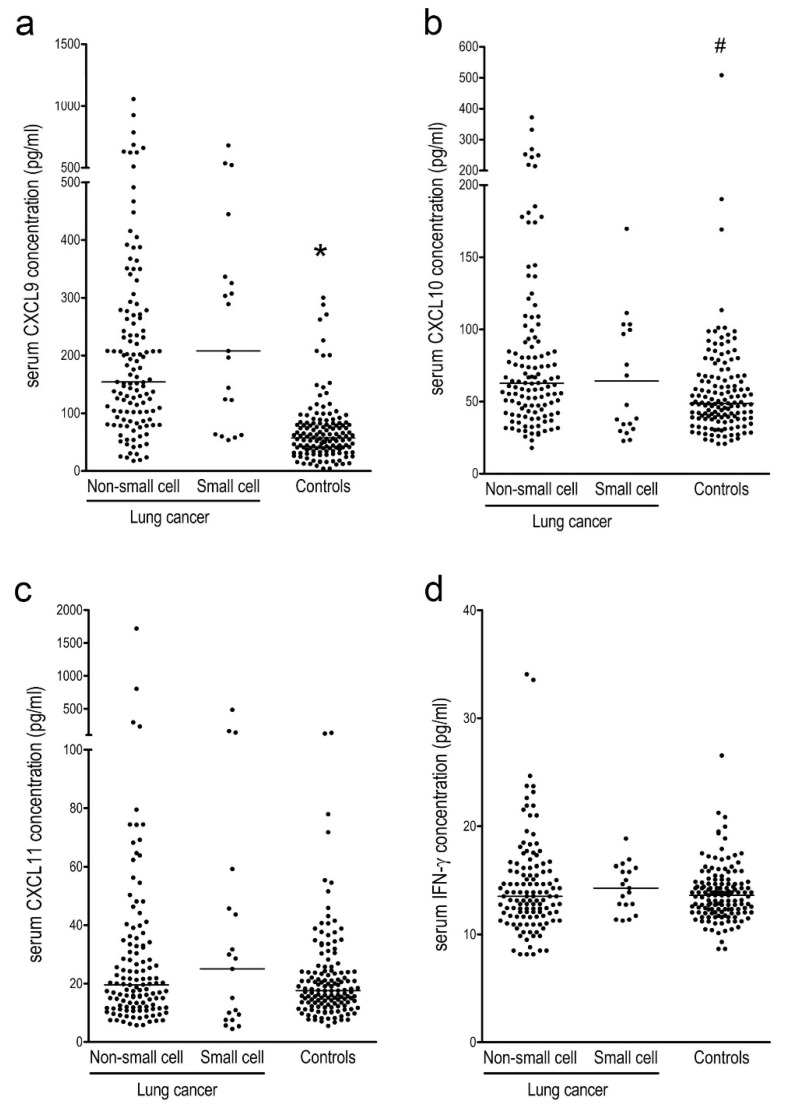
Serum levels of interferon (IFN)-γ-inducible chemokines CXCL9 (**a**), CXCL10 (**b**), and CXCL11 (**c**) and IFN-γ (**d**) levels in patients with lung cancer and healthy controls. Lung cancer patients are further divided into non-small cell and small cell lung cancers. * *p* < 0.0001 compared to both non-small cell carcinoma and small cell lung cancer. ^#^ *p* < 0.007 compared to non-small cell lung cancer.

**Figure 2:**
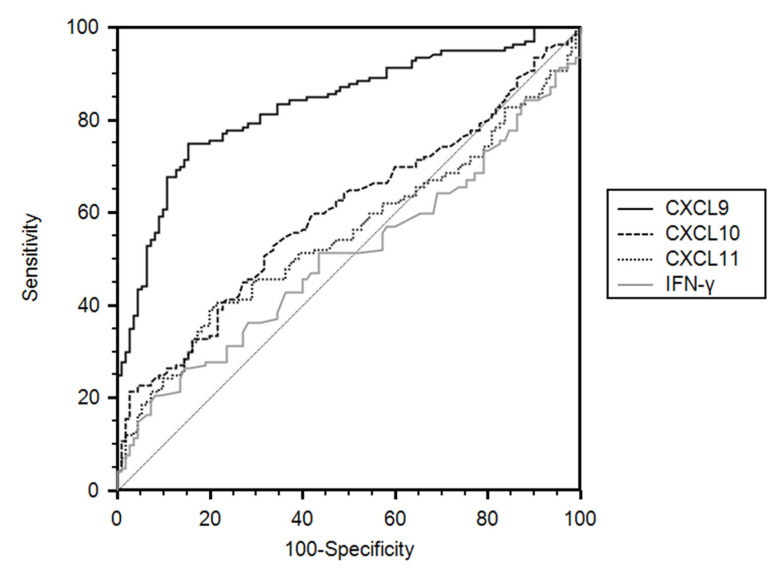
Receiver operating characteristics curves of interferon (IFN)-γ-inducible chemokines CXCL9, CXCL10, and CXCL11 and IFN-γ for differentiating all patients with lung cancer from the controls.

Serum levels of CXCL9 and CXCL10 were significantly higher in patients with NSCLC and SCLC than in controls. No significant differences in the serum levels of CXCL11 or IFN-γ between lung cancer patients and controls were observed (Figure 1a–d). Based on ROC analysis, the AUC (95% CI) of CXCL9 was 0.84 (0.80–0.88) for differentiating lung cancer patients from controls. With a cut-off value of 100.5 pg/mL, the sensitivity and specificity were 74.3% and 87.1%, respectively. Other markers showed only marginal discriminating power, with AUCs of 0.62 (0.56–0.68) for CXC10, 0.54 (0.48–0.60) for CXCL11, and 0.52 (0.46–0.58) for IFN-γ (Figure 2).

### 3.3. Serum IFN-γ-Inducible Chemokines and IFN-γ Levels in NSCLC and SCLC Patients

The serum levels of CXCL9, CXCL10, and CXCL 11 were significantly correlated with one another in all lung cancer and NSCLC patients, but the degree of correlation was relatively weak. The serum levels of IFN-γ were not correlated with the serum levels of CXL9, CXCL10, and CXCL11, respectively, in all lung cancer and NSCLC patients (Table S1).

The serum levels of IFN-γ-inducible chemokines and IFN-γ did not differ significantly between patients with NSCLC and SCLC. In patients with NSCLC, the levels of all the markers did not differ significantly among the pathologic types (Table S2; Figure S1).

In patients with NSCLC, there was no significant difference in marker serum levels between any of the stages except for a higher level of CXCL10 in stage IV patients versus those with stage I. However, the levels of all markers were significantly higher in stage IV NSCLC patients than in patients with stages I–III combined. In patients with SCLC, there were no significant differences in any markers between the limited and extensive stages (Table 2; Figures S2–S4).

### 3.4. Survival Analysis

**Figure 3:**
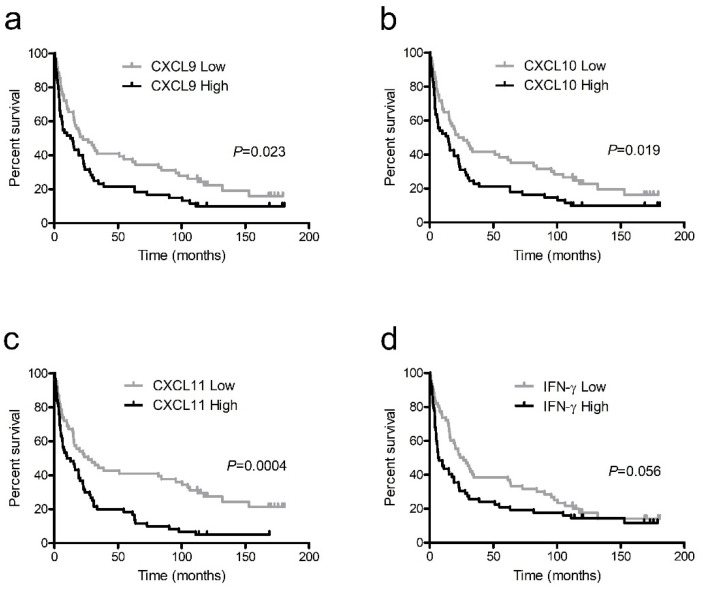
Kaplan–Meier survival curves for overall survival in non-small cell lung cancer patients with high and low levels of interferon (IFN)-γ-inducible chemokines CXCL9 (**a**), CXCL10 (**b**), and CXCL11 (**c**) and IFN-γ (**d**).

**Figure 4:**
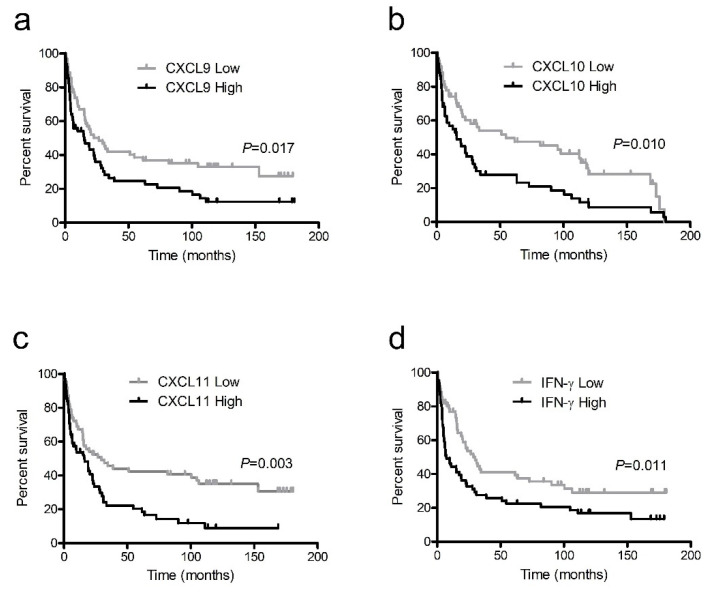
Kaplan–Meier survival curves for cancer-specific survival in non-small cell lung cancer patients with high and low levels of interferon (IFN)-γ-inducible chemokines CXCL9 (**a**), CXCL10 (**b**), and CXCL11 (**c**) and IFN-γ (**d**).

In patients with NSCLC, the mean overall survival period was 48.9 ± 5.6 months and the mean cancer-specific survival period was 57.4 ± 6.5 months. The serum levels of CXCL9, CXCL10, CXCL11, and IFN-γ were dichotomized into high and low groups according to the median values, 154.4 pg/mL, 62.9 pg/mL, 19.6 pg/mL, and 13.5 pg/mL, respectively, and used as prospective criteria to predict survival. Based on the Kaplan–Meier survival analysis, NSCLC patients with higher levels of all IFN-γ-inducible chemokines had significantly poorer outcomes than those with lower levels in terms of overall survival (Figure 3a–c) and cancer-specific survival (Figure 4a–c). Patients with lower IFN-γ levels tended to have better overall survival (Figure 3d) and significantly better cancer-specific survival compared to those with higher levels (Figure 4d).

Based on the multivariate Cox proportional hazards model, levels of CXCL9 and CXCL11 remained independent prognosticators for overall survival after controlling for significant covariates such as patient age and cancer stage from the univariate Cox model. For the multivariate Cox model for cancer-specific survival, CXCL9 showed significant results and CXCL11 showed a trend toward significance as independent prognosticators after controlling for the same covariates. CXCL10 and IFN-γ failed to show significant results in the multivariate survival analysis (Table 3).

In patients with SCLC, the survival analysis showed no significant differences for any markers (data not shown).

## 4. Discussion

Our results show that the IFN-γ-inducible chemokines are useful prognostic markers in NSCLC patients. In particular, CXCL9 showed the most significant results as an independent prognosticator both for overall survival and for cancer-specific survival. CXCL11 showed significant results for overall survival analysis, while CXCL10 and IFN-γ did not show any significant results in the multivariate survival analysis.

The IFN-γ-inducible chemokines had higher blood concentration levels and showed better clinical utility than IFN-γ. Our previous studies also showed a similar level of superiority for IFN-γ-inducible chemokines over IFN-γ in the blood samples of tuberculosis patients [30,31]. Among the IFN-γ-inducible chemokines, CXCL9 was the most prevalent and showed the most significant results, which also was the case for the tuberculosis studies [30,31].

In the Kaplan–Meier survival analysis and univariate Cox proportional hazards model, most of the markers showed significant results. However, after controlling for significant confounding variables, such as age and stage of disease in the multivariate Cox model, the significance level of the markers generally declined.

Patients with NSCLC showed a higher level of IFN-γ-inducible chemokines in stage IV than in stages I–III combined. Similar results were seen in a previous study where NSCLC patients with metastases showed higher levels of IFN-γ-inducible chemokines than those in earlier stages [26]. This phenomenon may reflect the decrease in significance observed for serum IFN-γ-inducible chemokines in the multivariate survival analysis. Previously, other types of malignancies were reported to show a relationship between stage progression or metastasis and an increased level of circulating IFN-γ-inducible chemokines: CXCL9 in nasopharyngeal cancer [23], uterine cervical cancer [32], and liver cancer [33]; CXCL10 in colorectal cancer [20]; and CXCL9 and CXCL10 in gastric cancer [34].

For diagnostic purposes, serum IFN-γ-inducible chemokines showed limited utility for discriminating between patients with lung cancer and controls, mainly because levels were relatively low in the early stages of lung cancer. Only CXCL9 showed moderate diagnostic potential for lung cancer, and a previous study showed similar results in lung cancer patients [26].

The higher expression levels of CXCR3 and IFN-γ-inducible chemokines on cancer tissues have been reported to be associated with poor survival in breast cancer [19], colon cancer [17], glioma [18], and lung cancer [35]. With regard to the prognostic implications of circulating IFN-γ-inducible chemokines, previous studies have shown that their increased blood levels are correlated with poor prognosis in various types of malignancies [20,21,22,23,24]. This suggests that circulating IFN-γ-inducible chemokines may preferentially interact with CXCR3 expressed on tumor cells, while the action of CXCR3 on immune cells may be less prominent. In addition, the differential actions of CXCR3 isoforms and the immune evasion of tumor cells may be associated with these phenomena. However, it is difficult to ascertain the exact mechanisms at this time.

Since the CXCR3/ligand axis is associated with Th1-cell-mediated immunity, a previous history of tuberculosis and other chronic diseases may potentially influence the blood levels of IFN-γ-inducible chemokines. However, simple blood concentrations of IFN-γ-inducible chemokines did not differ significantly based on history of chronic diseases (data not shown).

There are several limitations to our study. First, the number of patients was relatively small compared to the enrollment period since we included only patients whose outcomes were definitively identified and patient recruitment was not very active during some periods. However, the relatively long periods of follow-up may be a strength for the results of our survival analysis. Second, in patients with SCLC, a survival analysis and other detailed statistical analyses could not be presented due to the limited number of patients. Third, the responses to anti-cancer treatments were not incorporated into the survival analysis, since the treatment modalities and intensities were variable depending on the general status of patients, and the treatment responses were generally poor.

## 5. Conclusions

Measurements of blood IFN-γ-inducible chemokine levels have validity in clinical assessment of patients with lung cancer. In particular, they can be used as significant markers for predicting the survival of patients with NSCLC.

## Figures and Tables

**Table 1:** Demographic data.

	Lung Cancer	Control	*p*-Value
Number	144	140	
Age (years)	69 (61–75)	63 (58–69)	NS
Male	106 (73.6)	94 (67.1)	NS
Smoker	79 (54.9)	50 (35.7)	<0.01
History of diseases			
Tuberculosis	5	4	
Other malignancies	7	0	
Concomitant diseases			
Diabetes mellitus	5	0	
Liver cirrhosis	2	0	
IPF	3	0	
Cell types			
NSCLC	125 (86.8)		
Squamous	40 (27.8)		
Adenocarcinoma	68 (47.2)		
Large cell	4 (2.7)		
Others	13 (9.0)		
SCLC	19 (13.1)		
Stages			
NSCLC			
I	37 (25.7)		
II	13 (9.0)		
III	37 (25.7)		
IV	38 (26.4)		
SCLC			
Limited	9 (6.3)		
Extensive	10 (6.9)		
Treatment modalities			
NSCLC			
Surgical resection	72 (50.0)		
Chemotherapy alone	12 (8.3)		
Radiotherapy alone	20 (13.9)		
Chemoradiotherapy	12 (8.3)		
Palliative treatment	9 (6.2)		
SCLC			
Chemotherapy alone	5 (3.5)		
Radiotherapy alone	2 (1.4)		
Chemoradiotherapy	7 (4.9)		
Palliative treatment	5 (3.5)		

The data are presented as median (25th–75th percentile) or number (%). NSCLC = non-small cell lung cancer; IPF = idiopathic pulmonary fibrosis; SCLC = small cell lung cancer.

**Table 2:** Comparison of IFN-γ-inducible chemokines and IFN-γ, depending on the stage in NSCLC and SCLC.

Stage	No.	CXCL9 (pg/mL)	CXCL10 (pg/mL)	CXCL11 (pg/mL)	IFN-γ (pg/mL)
NSCLC					
I–III	87	149.2 (86.2–234.9)	58.5 (40.5–81.0)	17.0 (11.3–30.4)	12.8 (11.6–15.1)
I	37	158.4 (112.0–231.5)	50.9 (32.8–74.7)	15.8 (11.2–26.6)	12.8 (12.0–15.1)
II	13	133.9 (82.7–207.6)	66.8 (40.5–73.8)	17.8 (12.7–37.3)	12.8 (12.0–15.5)
III	37	125.8 (79.8–242.9)	60.0 (47.391.2)	20.2 (10.8–28.5)	13.2 (11.6–15.5)
IV	38	207.4 (102.5–349.8) *	79.0 (54.1–129.0) ^#,†^	22.9 (15.9–41.2) *	15.0 (13.1–17.7) *
SCLC					
Limited	9	170.3 (124.3–208.1)	60.0 (37.6–99.7)	21.8 (9.8–30.0)	13.9 (12.8–15.8)
Extensive	10	296.2 (62.3–336.5)	34.6 (29.6–68.1)	17.9 (7.5–59.3)	14.3 (12.8–15.8)

The data are presented as median (25th–75th percentile). * *p <* 0.05 and ^#^ *p* < 0.01 compared to stages I–III, respectively. ^†^ *p* < 0.05 compared to stage I. IFN = interferon; NSCLC = non-small cell lung cancer; SCLC = small cell lung cancer.

**Table 3:** Univariate and multivariate Cox proportional hazards model for overall and cancer-specific survival in patients with non-small cell lung cancer.

Variable	Univariate			Multivariate		
	HR	95% CI	*p*-Value	HR	95% CI	*p*-Value
Overall survival						
Age (<60/≥60)	1.98	1.22–3.21	<0.001	1.05	1.02–1.07	<0.0001
Sex (male/female)	0.86	0.56–1.34	0.513			
Smoking (smoker/non-smoker)	0.85	0.58–1.25	0.402			
Histology (adenocarcinoma/squamous/others)	2.86	0.60–1.04	0.092			
Stage (I/II/III/IV)	2.20	1.79–2.70	<0.0001	2.26	1.84–2.78	<0.0001
CXCL9 (High/Low)	1.62	1.10–2.40	0.015	1.82	1.22–2.70	0.003
CXCL10 (High/Low)	1.90	1.28–2.83	0.002	1.36	0.89–2.07	0.146
CXCL11 (High/Low)	1.82	1.23–2.71	0.003	1.70	1.12–2.59	0.013
IFN-γ (High/Low)	1.43	0.97–2.11	0.068	1.32	0.88–1.98	0.185
Cancer-specific survival						
Age (<60/≥60)	1.71	1.03–2.82	0.037	1.05	1.02–1.07	<0.0001
Sex (male/female)	0.926	0.58–1.47	0.744			
Smoking (smoker/non-smoker)	0.81	0.54–1.23	0.326			
Histology (adenocarcinoma/ squamous/others)	0.89	0.66–1.19	0.427			
Stage (I/II/III/IV)	2.48	1.70–3.13	<0.0001	2.68	2.12–3.38	<0.0001
CXCL9 (High/Low)	1.68	1.11–2.54	0.015	1.91	1.25–2.93	0.003
CXCL10 (High/Low)	1.98	1.29–3.03	0.002	1.30	0.83–2.05	0.25
CXCL11 (High/Low)	1.76	1.15–2.68	0.009	1.50	0.96–2.33	0.074
IFN-γ (High/Low)	1.62	1.07–2.46	0.021	1.44	0.93–2.23	0.106

HR = hazard ratio; CI = confidence interval; IFN = interferon.

## Data Availability

Not applicable.

## Supplementary Materials

The following are available online at https://www.mdpi.com/article/10.3390/ijerph18179345/s1: Figure S1: title. Table S1: Correlation analysis among the serum levels of the markers in patients with lung cancer. Table S2: Comparison of serum levels of IFN-γ-inducible chemokines and IFN-γ depending on the histopathologic types of lung cancer. Figure S1. Comparison of the serum levels of IFN-γ-inducible chemokines CXCL9, CXCL10, and CXCL11 and IFN-γ levels in patients with lung cancer depending on the histopathologic types. Figure S2. Comparison of serum levels of IFN-γ-inducible chemokines CXCL9, CXCL10, and CXCL11 and IFN-γ levels depending on the stage in patients with non-small cell lung cancer. Figure S3. Comparison of serum levels of IFN-γ-inducible chemokines CXCL9, CXCL10, and CXCL11 and IFN-γ levels between stage I–III and stage IV of non-small cell lung cancer. Figure S4. Comparison of serum levels of IFN-γ-inducible chemokines CXCL9, CXCL10, and CXCL11 and IFN-γ levels between limited and extensive stages of small cell lung cancer.
