# Supplementary material for: Interferon-γ-Inducible Chemokines as Prognostic Markers for Lung Cancer
Source: Int J Environ Res Public Health. 2021 Sep 4;18(17):9345. doi: 10.3390/ijerph18179345 (PMC8431216; doi:10.3390/ijerph18179345)
Supplement: Supplementary file 1 [file ijerph-18-09345-s001.zip › ijerph-1359199-supplementary.pdf]

International Journal of
Environmental Research and Public Health

MDPI

Supplementary Data

Table S1. Correlation Analysis among the Serum Levels of the Markers in Patients with Lung Cancer

	CXCL9	CXCL10	CXCL11	IFN-γ
	All patients with lung cancer			
CXCL9	1			
CXCL10	0.39*	1		
CXCL11	0.33*	0.41*	1	
IFN-γ	0.06	0.43	0.10	1
	Patients with NSCLC			
CXCL9	1			
CXCL10	0.42*	1		
CXCL11	0.33*	0.49*	1	
IFN-γ	0.01	0.41	0.10	1

Values are Spearman Correlation Coefficients (r_s)
*p < 0.01
IFN = interferon; NSCLC = non-small cell lung cancer

Table S2. Comparison of Serum Levels of IFN-γ-Inducible Chemokines and IFN-γ Depending on the Histopathologic Types of Lung Cancer

	NSCLC				SCLC (N = 19)
	Squamous (N = 40)	Adenocarcinoma (N = 68)	Large cell (N = 4)	Others (N = 13)	
CXCL9	186.7 (127.3-260.7)	133.8 (80.2-233.2)	127.8 (92.9-152.9)	306.4 (264.8-466.9)	208.1 (63.6-336.5)
CXCL10	65.9 (44.5-103.6)	56.6 (42.2-80.6)	41.0 (38.0-94.5)	81.5 (69.0-109.4)	43.1 (31.0-99.7)
CXCL11	21.4 (15.1-35.7)	17.4 (11.4-28.1)	14.3 (10.1-18.0)	32.8 (16.3-56.3)	25.0 (7.6-45.7)
IFN-γ	12.8 (11.1-15.8)	13.5 (11.7-15.9)	14.1 (12.4-17.5)	14.7 (12.4-17.5)	14.3 (12.8-16.2)

The data are presented as median (25 percentile - 75 percentile) pg/ml.
IFN = interferon; NSCLC = non-small cell lung cancer; SCLC = small cell lung cancer.

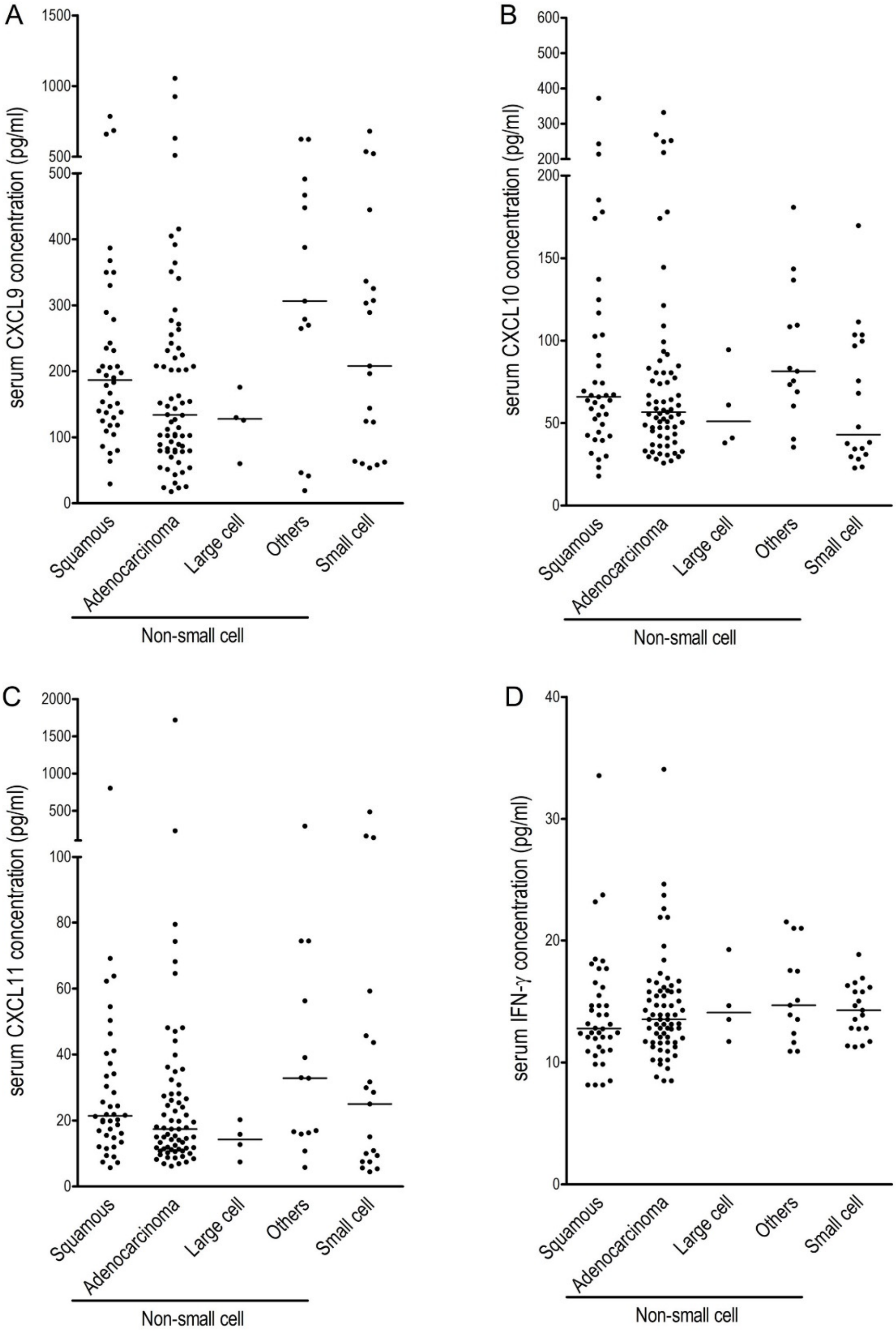

Figure S1. Comparison of the serum levels of interferon (IFN)-γ-inducible chemokines CXCL9 (A), CXCL10 (B), and CXCL11 (C) and IFN-γ (D) levels in patients with lung cancer depending on the histopathologic types.

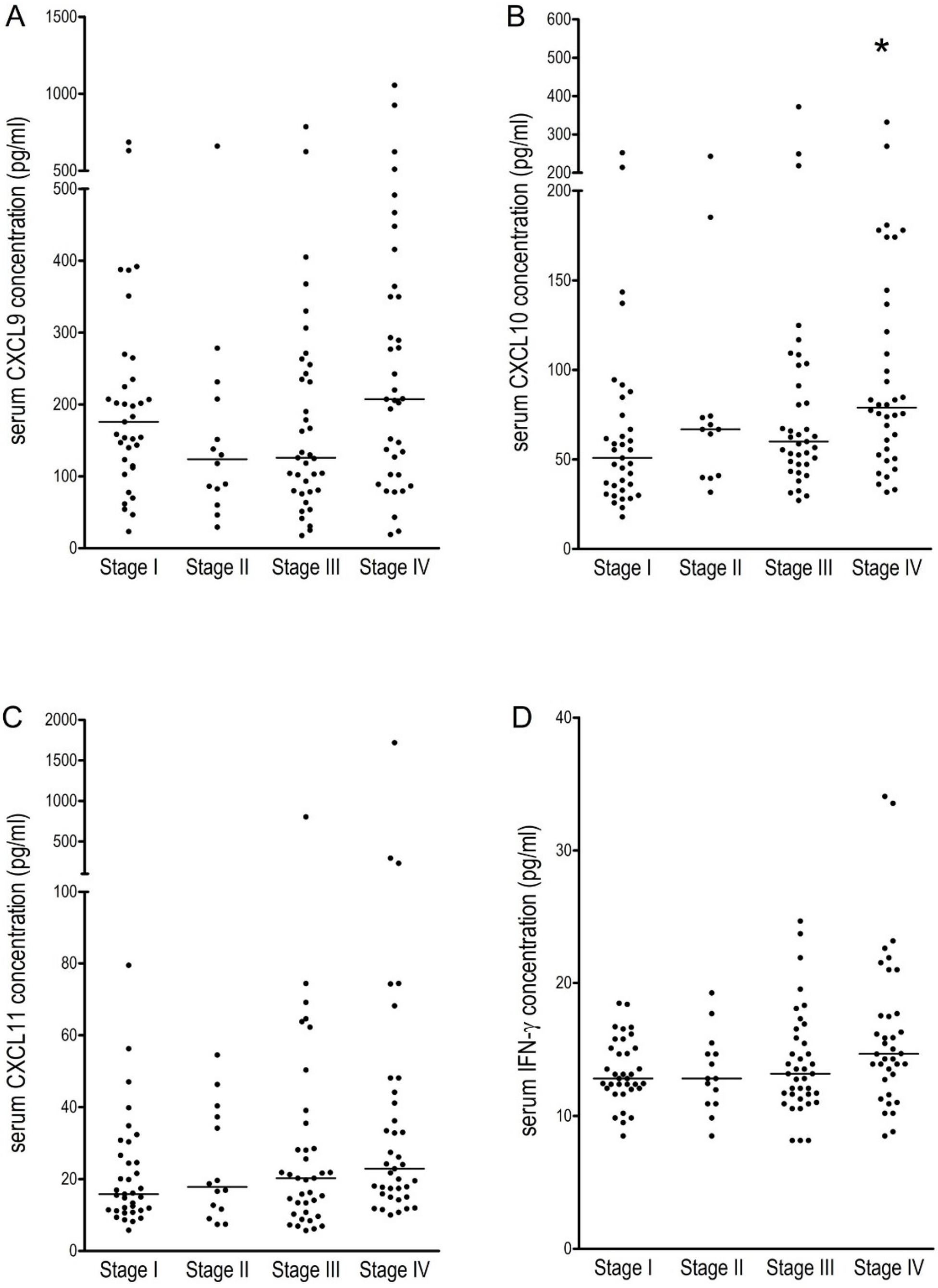

Figure S2. Comparison of serum levels of interferon (IFN)-γ-inducible chemokines CXCL9 (A), CXCL10 (B), and CXCL11 (C) and IFN-γ (D) levels depending on the stage in patients with non-small cell lung cancer. The stages are simplified to four stages due to limited number of the patients.

*$p < 0.05$ compared to Stage I

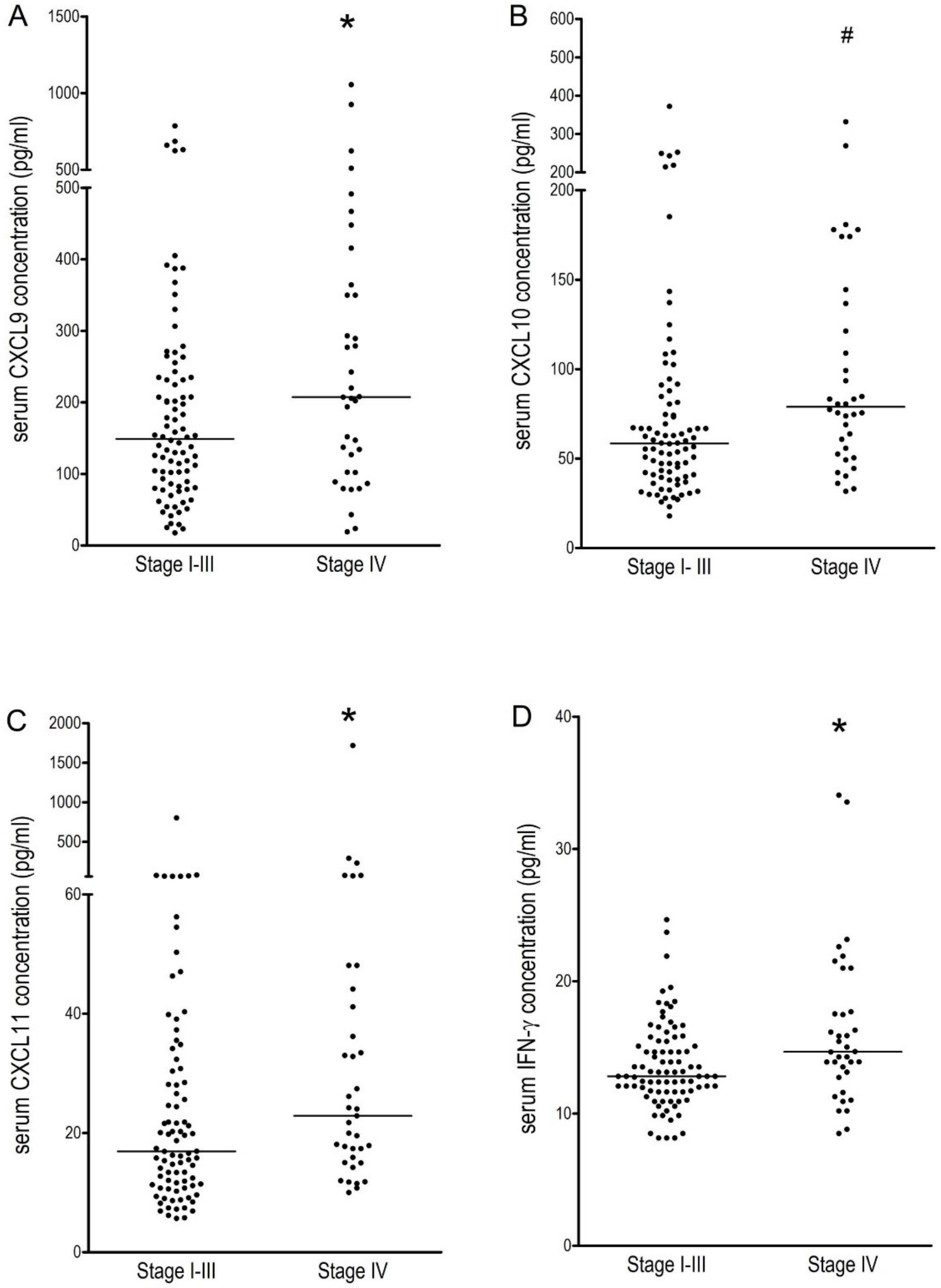

Figure S3. Comparison of serum levels of interferon (IFN)-γ-inducible chemokines CXCL9 (A), CXCL10 (B), and CXCL11 (C) and IFN-γ (D) levels between stage I-III and stage IV of non-small cell lung cancer.
*$p < 0.05$ and #$p < 0.01$ compared to stages I–III, respectively.

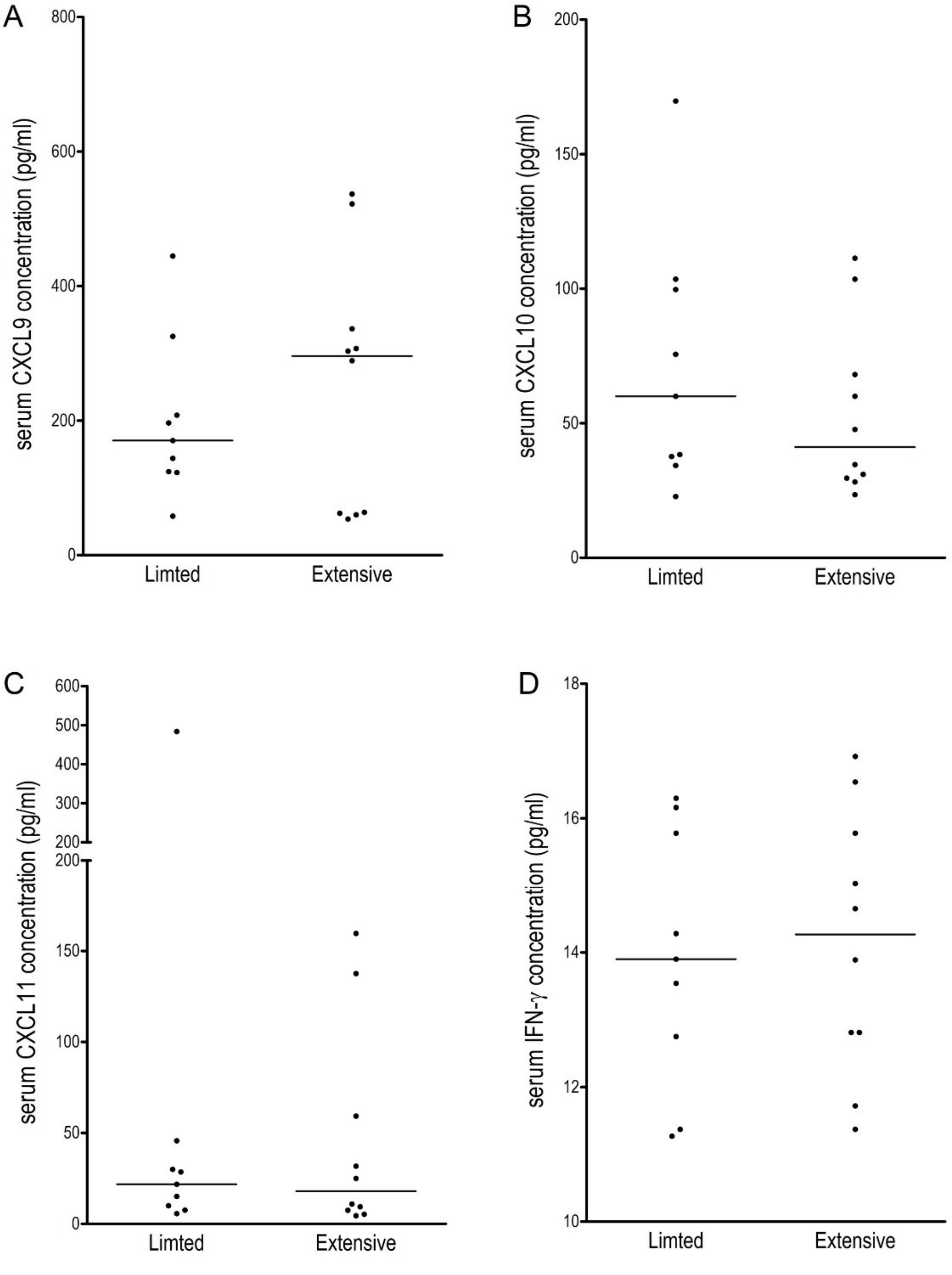

Figure S4. Comparison of serum levels of interferon (IFN)-γ-inducible chemokines CXCL9 (A), CXCL10 (B), and CXCL11 (C) and IFN-γ (D) levels between limited and extensive stages of small cell lung cancer.
